# Mobile Phone Addiction and Academic Procrastination Negatively Impact Academic Achievement Among Chinese Medical Students

**DOI:** 10.3389/fpsyg.2021.758303

**Published:** 2021-11-23

**Authors:** Jing Tian, Ji-yang Zhao, Jia-ming Xu, Qing-lin Li, Tao Sun, Chen-xi Zhao, Rui Gao, Li-yan Zhu, Hai-chen Guo, Li-bin Yang, De-pin Cao, Shu-e Zhang

**Affiliations:** ^1^Department of Health Management, School of Health Management, Harbin Medical University, Harbin, China; ^2^Party Committee Office, Chengdu Second People’s Hospital, Chengdu, China; ^3^Department of Health Management, School of Medicine, Hang Zhou Normal University, Hangzhou, China

**Keywords:** medical students, academic achievement, mobile phone addiction, academic procrastination, medical education

## Abstract

The problem of mobile phone addiction and academic procrastination among medical students has been widely acknowledged. This study aimed to explore the influence of demographic factors on mobile phone addiction, academic procrastination, and academic achievement among medical students. Further, it investigated the association between mobile phone addiction, academic procrastination, and academic achievement. This cross-sectional study was conducted between May and June 2019. A total of 3 511 medical students participated in an online questionnaire survey (effective response rate = 81.7%). Demographic factors, the Scale of Academic Achievement, the short scale of the Mobile Phone Problem Use (MPPUS-10), and the Academic Procrastination Scale–Short (APS-S) were used. Hierarchical multiple regression analysis revealed that the average scores for academic procrastination, mobile phone addiction, and academic achievement were 2.66 ± 0.91, 5.13 ± 1.53, and 4.51 ± 0.71, respectively. Moreover, there were significant differences in gender, grade, leadership experience, and family monthly income across mobile phone addiction, academic procrastination, and academic achievement. Mobile phone addiction was negatively associated with learning dedication, learning performance, and relationship facilitation. Academic procrastination was negatively associated with learning dedication, learning performance, relationship facilitation, and objective achievement. Mobile phone addiction and academic procrastination was revealed as prevalent among Chinese medical students, and negatively influences their academic achievement. It is critical to establish a more efficient learning environment for Chinese medical students to minimize the negative impact of mobile phone addiction and academic procrastination.

## Introduction

Medical education is essential for promoting the development of healthcare systems worldwide. Its primary objective is to train medical personnel to provide high-quality services to the public during their careers. Academic learning in medical school is crucial for students to acquire the knowledge and skills to become qualified doctors. Additionally, most countries lack talented and qualified physicians, resulting in a sustained demand to cultivate qualified medical students. Academic achievement is defined as the sum of medical students’ learning consequences, attitudes, and behaviors (Yanfei et al., 2011). It comprises two aspects: behavioral performance and objective achievement. Academic achievement is often regarded as an index for evaluating training outcomes in theoretical and practical courses (Özcan et al., 2019). Studies suggest that academic achievement is associated with learning strategies (Rugutt, 2005), outcome expectations (Nabizadeh et al., 2019), thinking skills, learning styles (Shirazi and Heidari, 2019), lifestyle (Heidari, 2017), self-esteem (Jirdehi et al., 2018), family support (Abdulghani et al., 2014), and social and psychological factors (Džubur et al., 2020). Conversely, academic procrastination is a psychological factor, which is negatively correlated with academic achievement among college students as reported by a study done on Turkish medical students (Karatas, 2015). Moreover, with the rapid development of the Internet, mobile phone addiction has emerged as an important factor affecting students’ academic achievement (Ibrahim et al., 2018). Considering cross-cultural differences, continuous attention should be given to academic achievement and its influencing factors among medical students in China.

According to the 47th Statistical Report on Internet Development in China, ‘‘As of December 2020, the number of online surfers by cellphone in China has reached 986 million.’’^1^ Simultaneously, the penetration rate of mobile phones in Chinese university campuses is close to 100% (Rong and Hong, 2018). Mobile phones have proven useful for medical students as practical learning tools that enable them to “learn anywhere” (Payne et al., 2012). Moreover, mobile phones have a wide range of functionality in elevating the accessibility of learning and realizing equal opportunities for education. However, excessive and problematic use has caused adverse effects on the learning behavior of medical students (Ibrahim et al., 2018). Mobile phone addiction is defined as the uncontrolled use of mobile phones in inappropriate or harmful situations (Bianchi and Phillips, 2005) and is common among contemporary college students (Gao et al., 2018). Studies have found that adolescents and young people have been exposed to mobile phone addiction, which hinders their academic performance (Lepp et al., 2015; Xavier et al., 2018). Moreover, mobile phone addiction is negatively associated with academic performance among university students globally, and high-frequency mobile phone users spent less time on academic attention, interest, and investment (Amez and Baert, 2020). The study of medicine presents unique challenges with numerous courses and immense amounts of necessary scientific knowledge and practical skills, requiring medical students to dedicate more time and energy to studying it (Reed et al., 2014; Yeh and Park, 2015). However, in China, medical students with mobile phone addiction dedicate their limited energy resources to their mobile phones, resulting in decreased academic performance and a series of negative outcomes that potentially impact the medical profession (Siyu et al., 2020). Based on the characteristics of medical students, this study aimed to investigate the relationship between mobile phone addiction and academic achievement among medical students in China.

Procrastination is a common phenomenon, often occurring in a pragmatic and technologically advanced society (Steel, 2007), causing poor mental health, diminished success, increased stress, and reduced well-being (Jaffe, 2013; Glick and Orsillo, 2015). Academic procrastination is a type of situational procrastination (Karimi Moonaghi and Baloochi Beydokhti, 2017), and is defined as an initiative delay in the learning process and intended course (Steel, 2007). Although many studies have explored academic procrastination in different educational settings, the university context has been the most common (Karimi Moonaghi and Baloochi Beydokhti, 2017). For example, the prevalence of academic procrastination was 68% among college students in Iran (Rafii et al., 2014) and between 70 and 80% in Sweden (Rozental and Carlbring, 2014). Recent research has focused on academic procrastination and academic achievement among college students, finding a negative correlation between them (Karatas, 2015). Moreover, medical students are more prone to academic procrastination than college students (Forough et al., 2015; Hayat et al., 2020). Medical students must manage course schedules, teaching content, and academic tasks; thus, they are subjected to longer schooling, containing multiple courses and academic tasks. They therefore experience heavy academic burdens, and high pressure (Ross et al., 2010; Kötter et al., 2017), resulting in negative emotions and academic procrastination (Artino et al., 2012). In China, academic procrastination occurs more among medical than non-medical college students, and academic procrastination is further influenced by gender, life satisfaction, and anxiety among this population (Yao et al., 2021). However, few studies have directly explored the relationship between academic procrastination and academic achievement among Chinese medical students.

Therefore, this study aimed to explore the influence of demographic factors on mobile phone addiction, academic procrastination, and academic achievement among medical students. We further explored the association between mobile phone addiction, academic procrastination, and academic achievement.

## Materials and Methods

### Study Design and Procedures

A cross-sectional online survey was conducted in Heilongjiang Province, China, ensuring time-effectiveness, cost-effectiveness, and accessibility of the research (Chang and Vowles, 2013). Four medical colleges were selected as survey sites: Harbin Medical University, Jiamusi Medical College, Mudanjiang Medical University, and Qiqihar Medical University. The colleges differed in size, major setting, and academic competence. Moreover, medical students from the selected colleges came from different provinces in China. We used a multistage stratified convenient sampling method; participants from different classes and grades were randomly selected. The procedures of this survey were approved by the Ethics Committee of the Institutional Review Board of Harbin Medical University before starting the online investigations and observed throughout the questionnaire survey process. Before the formal investigation commenced, Harbin Medical University was selected as our pilot survey site, and 300 questionnaires were issued and collected via the Internet. These pilot data were used for questionnaire improvement and hence were excluded from the main analysis. Finally, we consulted medical education experts, college administrators, and medical teachers (a total of four experts); based on their feedback, the questionnaire was modified.

### Data Collection

The minimum sample size was calculated as 1,824 medical students, according to the standard requirements and calculation method recommended by Zhou et al. (2017). Furthermore, considering the response rate and data quality, the recommended sample size was expanded to 5,000. The survey was conducted between May and June 2019. Before the investigation commenced, we obtained informed consent from the participants. The online questionnaire survey was hosted by ‘‘Questionnaire Star.’’^2^ Each medical student who agreed to participate in the study was informed of the topic and content of the survey. The questionnaire URL was distributed to participants to complete in their spare time; each participant could only reply once. We carefully monitored the questionnaire collection process and effectively managed the data on Questionnaire Star. Additionally, we checked the collected questionnaires daily for quality control purposes. Such a survey method has been successfully employed in other completed studies (Zhang et al., 2018; Shi et al., 2021). Questionnaires were distributed to a total of 5,921 participants, and 4,297 questionnaires were successfully returned. The inclusion criteria were: (1) medical students studying at the selected medical college, (2) voluntarily and truthfully responding to the online questionnaire survey, and (3) having complete answers. The exclusion criteria were: (1) taking less than 8 minutes (which was confirmed as the minimum answering time in the preliminary survey) to answer the questionnaire, and (2) answering quality control questions incorrectly (for example, “Did you fill out the questionnaire carefully?”). Ultimately, 3 511 questionnaires were valid (effective response rate = 81.7%).

### Measures

#### Demographic Characteristics

Information on five demographic characteristics was collected from a self-designed questionnaire, comprising sex, region, year of study, leadership experience, and family income. Region was divided into two categories: “rural” and “urban.” The year of study was recorded as a continuous variable, from 1 to 5. Leadership experience was divided into “student leaders” and “ordinary students.” Options for family income included “≤ ¥5,000,” “¥5,001–¥10,000,” “¥10,001–¥20,000,” and “≥ ¥20,001.”

#### Academic Achievement

Academic achievement was measured using 19 items developed by Yanfei et al. (2011). Academic achievement was divided into two sides: behavior performance and objective achievement. Behavior performance included three subscales: learning performance (6 items), relationship facilitation (6 items), and learning dedication (3 items), totaling to 15 items. Each item of behavioral performance was rated on a 6-point Likert scale (1 = *strongly unapplicable*, and 6 = *strongly applicable*). Objective achievement comprised four items for the participants’ self-evaluation of achievement (recreational and sports activities, moral education, intellectual education, and total score) using a 5-point Likert scale. The higher the participants’ self-evaluation, the higher their subjective achievement. Cronbach’s alpha for the overall scale was 0.936 and those for the subscales were 0.895 (learning performance), 0.907 (relationship facilitation), 0.902 (learning dedication), and 0.874 (objective achievement).

#### Mobile Phone Addiction

Mobile phone addiction was measured using the Chinese adaptation (Hongjuan et al., 2017) of the short scale of Mobile Phone Problem Use (MPPUS-10) (Milena et al., 2015). The MPPUS-10 is a 10-item scale consisting of five dimensions: craving (1 item), negative life consequences (2 items), peer acceptance (1 item), withdrawal (3 items), and loss of control (3 items). Answers are recorded on a 5-point Likert scale (1 = *strongly unapplicable* to 5 = *strongly applicable*), with higher scores indicating higher mobile phone addiction. The MPPUS-10 has demonstrated adequate reliability and validity in previous studies in China (Hongjuan et al., 2017; Jianfang, 2018). For example, Hongjuan et al. (2017) used the MPPUS-10 to investigate middle school students in Beijing, and Cronbach’s alpha was 0.87 (Hongjuan et al., 2017). Additionally, the MPPUS-10 was used for stratified sampling to investigate problematic mobile phone use among high school students in China, where Cronbach’s alpha was 0.83 (Jianfang, 2018). In this study, Cronbach’s alpha was 0.825.

#### Academic Procrastination

The Academic Procrastination Scale–Short (APS-S) was adopted to measure the severity of the effect of procrastination on students’ academic tasks (Yockey, 2016). It has a total of five items, with each item examining the respondent’s learning experience. Answers were rated on a 5-point Likert scale (1 = *totally agree* and 5 = *totally disagree*). Higher scores indicated a greater tendency to procrastinate on academic tasks. Cronbach’s alpha was 0.901 in this study.

### Statistical Analysis

IBM SPSS Statistics 23.0 was used to analyze the data. Participants’ demographic characteristics (sex, region, grade, leadership experience, and family income [RMB]) were collected to provide sample information. Variance analysis was used to test for associations between demographic characteristics and mobile phone addiction, academic procrastination, and academic achievement. Multiple linear hierarchical regression analysis was performed to examine the relationships among the variables. *P* < 0.05 (two-tailed significance test) was considered significant for all statistical tests in this study.

## Results

### Demographics and Characteristics

The percentages of participants who were female, lived in urban areas, and were student leaders were 65.99, 54.09, and 35.46%, respectively. There were 30.82, 28.51, 22.67, 6.69, and 11.31% participants in grades one, two, three, four, and five, respectively. Regarding participants’ family monthly income levels, 41.81, 48.45, 9.00, and 0.74% participants indicated incomes ≤ ¥5,000, ¥5,001–10,000, ¥10,001–20,000, and ≥ ¥20,001, respectively.

### Descriptive Statistics

As shown in Table 1, descriptive statistical analysis was used to analyze the mean distribution of mobile phone addiction, academic procrastination, and the four dimensions of academic achievement. This included objective achievement, relationship facilitation, learning performance, and learning dedication.

**TABLE 1:** Means (*M*) and standard deviations (*SD*) of the basic variables and dimensional inventory of academic achievement (*n* = 3,511).

	*M* ± *SD*	Min–Max
Mobile phone addiction	5.13 ± 1.53	1–10
Academic procrastination	2.66 ± 0.91	1–5
Objective achievement	3.42 ± 0.77	1–5
Relationship facilitation	4.62 ± 0.77	1–6
Learning performance	4.52 ± 0.77	1–6
Learning dedication	4.29 ± 0.92	1–6

### Difference Between Participants’ Characteristics and Scores of Multiple Variables

Scores for learning dedication differed significantly according to participants’ demographics, including sex, grade, and leadership experience. The descriptive associations between participants’ characteristics and mobile phone addiction, academic procrastination, objective achievement, relationship facilitation, and learning performance scores are shown in Table 2.

**TABLE 2:** Variance analysis and description of each variable.

					Academic achievement			
Variables		*N* (%)	Mobile phone addiction	Academic procrastination	Learning dedication	Learning performance	Relationship facilitation	Objective achievement
			*M* ± *SD*	*M* ± *SD*	*M* ± *SD*	*M* ± *SD*	*M* ± *SD*	*M* ± *SD*
Gender	Male	1,194 (34.01)	5.24 ± 1.64	2.68 ± 0.98	4.35 ± 1.00	4.51 ± 0.86	4.64 ± 0.84	3.37 ± 0.84
	Female	2,317 (65.99)	5.07 ± 1.48	2.64 ± 0.88	4.27 ± 0.87	4.52 ± 0.72	4.60 ± 0.73	3.44 ± 0.73
	*t/F*		9.536**	1.624	6.175*	0.169	1.774	6.585*
	*P*		0.002	0.203	0.013	0.681	0.183	0.010
Region	Rural	1,612 (45.91)	5.16 ± 1.54	2.66 ± 0.90	4.28 ± 0.89	4.53 ± 0.73	4.60 ± 0.75	3.41 ± 0.76
	Urban	1,899 (54.09)	5.11 ± 1.53	2.65 ± 0.93	4.30 ± 0.94	4.51 ± 0.80	4.63 ± 0.79	3.43 ± 0.78
	*t/F*		1.032	0.088	0.425	0.833	0.918	-0.142
	*P*		0.310	0.767	0.515	0.361	0.338	0.706
Grade	One	1,082 (30.82)	5.16 ± 1.49	2.65 ± 0.92	4.36 ± 0.87	4.56 ± 0.74	4.70 ± 0.73	3.42 ± 0.72
	Two	1,001 (28.51)	5.14 ± 1.51	2.60 ± 0.88	4.31 ± 0.93	4.54 ± 0.79	4.62 ± 0.79	3.42 ± 0.79
	Three	796 (22.67)	5.21 ± 1.56	2.71 ± 0.93	4.19 ± 0.99	4.47 ± 0.810	4.52 ± 0.80	3.38 ± 0.81
	Four	235 (6.69)	4.90 ± 1.55	2.63 ± 0.90	4.24 ± 0.89	4.40 ± 0.73	4.54 ± 0.72	3.40 ± 0.79
	Five	397 (11.31)	5.00 ± 1.63	2.70 ± 0.94	4.32 ± 0.86	4.54 ± 0.73	4.65 ± 0.75	3.49 ± 0.80
	*t/F*		2.760*	1.853	4.347**	3.048*	7.052**	1.292
	*P*		0.026	0.116	0.002	0.016	<0.001	0.271
Leadership experience	Student leaders	1,245 (35.46)	5.12 ± 1.55	2.58 ± 0.94	4.44 ± 0.94	4.62 ± 0.79	4.81 ± 0.77	3.67 ± 0.75
	Ordinary students	2,266 (64.54)	5.14 ± 1.53	2.70 ± 0.90	4.21 ± 0.89	4.47 ± 0.75	4.51 ± 0.75	3.28 ± 0.75
	*t/F*		0.173	13.154**	49.398**	30.673**	126.183**	225.279**
	*P*		0.677	<0.001	<0.001	<0.001	< 0.001	<0.001
Family monthly income (RMB)	≤5,000	1,468 (41.81)	5.13 ± 1.55	2.66 ± 0.94	4.28 ± 0.92	4.50 ± 0.77	4.60 ± 0.78	3.39 ± 0.77
	5,001–10,000	1,701 (48.45)	5.14 ± 1.51	2.64 ± 0.89	4.29 ± 0.89	4.51 ± 0.75	4.62 ± 0.75	3.43 ± 0.76
	10,001–20,000	316 (9.00)	5.08 ± 1.57	2.67 ± 0.94	4.34 ± 1.03	4.62 ± 0.84	4.66 ± 0.83	3.49 ± 0.82
	≥20,001	26 (0.74)	4.90 ± 1.85	2.80 ± 1.12	4.74 ± 0.94	4.69 ± 0.80	4.71 ± 0.88	3.77 ± 0.89
	*t/F*		0.309	0.405	2.552	2.558	0.519	3.672*
	*P*		0.819	0.749	0.054	0.051	0.669	0.012

*P < 0.05, **P < 0.01, Pearson Correlation is significant at the 0.01 level (2-tailed).

### Multiple Linear Hierarchical Regression Analysis Models for Participants’ Academic Achievement

Academic achievement was negatively correlated with mobile phone addiction (*r* = −0.780, *p* < 0.01) and academic procrastination (*r* = −0.285, *p* < 0.01). Additionally, mobile phone addiction was positively correlated with academic procrastination (*r* = 0.457, *p* < 0.01). Subsequently, a multiple linear hierarchical regression analysis was performed to examine the influence of mobile phone addiction and academic procrastination on academic achievement after controlling for the effects of the demographic variables.

Table 3 represents the influence of mobile phone addiction on academic achievement, and Table 4 shows the influence of academic procrastination on academic achievement. We found that mobile phone addiction was significantly negatively associated with learning dedication (β = −0.080, *p* < 0.01), learning performance (β = −0.112, *p* < 0.01), and relationship facilitation (β = −0.033, *p* < 0.05). Meanwhile, academic procrastination was significantly negatively related to learning dedication (β = −0.220, *p* < 0.01), learning performance (β = −0.322, *p* < 0.01), relationship facilitation (β = −0.171, *p* < 0.01), and objective achievement (β = −0.154, *p* < 0.01).

**TABLE 3:** Influence of mobile phone addiction on academic achievement.

Variables	*M*_1_ (β)	Academic achievement			
		*M*_2_ (β)	*M*_3_ (β)	*M*_4_ (β)	*M*_5_ (β)
Control variables					
Gender	−0.039*	−0.043*	0.004	–0.020	0.049**
Grade	–0.027	–0.030	–0.025	−0.033*	0.033*
Region	–0.005	–0.006	−0.036*	–0.001	–0.027
Family monthly income	0.023	0.022	0.042*	0.012	0.054**
Leadership experience	−0.115**	−0.114**	−0.092**	−0.183**	−0.249**
Predictor variable					
Mobile phone addiction		−0.080**	−0.112**	−0.033*	–0.018
*F*	11.938**	13.788**	14.312**	22.667**	41.658**
*R* ^2^	0.017**	0.021**	0.022**.	0.036*	0.065
Δ*R*^2^	0.015**	0.023**	0.024**	0.037*	0.067

*M_1_: control variables, including gender, grade, region, family monthly income, and leadership experience. M_2_: explains the influence of mobile phone addiction on learning dedication. M_3_: explains the influence of mobile phone addiction on learning performance. M_4_: explains the influence of mobile phone addiction on relationship facilitation. M_5_: explains the influence of mobile phone addiction on objective achievement.*

*P < 0.05, **P < 0.01, Pearson Correlation is significant at the 0.01 level (2-tailed).

**TABLE 4:** Influence of academic procrastination on academic achievement.

Variables	*M*_6_ (β)	Academic achievement			
		*M*_7_ (β)	*M*_8_ (β)	*M*_9_ (β)	*M*_10_ (β)
Control variables					
Gender	−0.039*	−0.044**	0.003	–0.023	0.046**
Grade	–0.027	–0.023	–0.015	–0.029	0.036*
Region	–0.005	–0.005	−0.035*	–0.001	–0.027
Family monthly income	0.023	0.024	0.044**	0.012	0.054**
Leadership experience	−0.0115**	−0.102**	−0.073**	−0.173**	−0.240**
Predictor variable					
Academic procrastination		−0.220**	−0.322**	−0.171**	−0.154**
*F*	11.938**	40.628**	75.775**	40.919**	57.715**
*R* ^2^	0.017**	0.063**	0.113**	0.064**	0.088**
Δ*R*^2^	0.015**	0.065**	0.115**	0.065**	0.090**

*M_6_: control variables, including gender, grade, region, family monthly income, and leadership experience. M_7_: explains the influence of academic procrastination on learning dedication. M_8_: explains the influence of academic procrastination on learning performance. M_9_: explains the influence of academic procrastination on relationship facilitation. M_10_: explains the influence of academic procrastination on objective achievement.*

*P < 0.05, **P < 0.01, Pearson Correlation is significant at the 0.01 level (2-tailed).

## Discussion

This study investigated the association between academic procrastination, mobile phone addiction, and academic achievement among Chinese medical students. Further, the scores for academic procrastination and academic achievement were higher than those reported by previous studies examining college students from non-medical universities in China (Haiqin et al., 2013; Jiajia et al., 2014). These differences may be attributed to different survey tools and variations in target populations, such as medical versus non-medical students. Moreover, the score for mobile phone addiction was higher than that reported among adults aged 18–34 years using MPPUS-10 in a Lebanese study (Marc et al., 2018). Chinese medical students are influenced by professional and environmental factors and are faced with immense academic pressure and strict standards; thus, they are prone to social anxiety and are vulnerable to mobile phone addiction (Linlin et al., 2015). These findings suggest that academic procrastination and mobile phone addiction levels among Chinese medical students are above the average and should be given increased attention.

### Influence of Sociodemographic Characteristics on Mobile Phone Addiction, Academic Procrastination, and Academic Achievement

Sex, grade, leadership experience, and family monthly income were found to affect male medical students. This may lead to anxiety and poor sleep quality, which may subsequently cause higher mobile phone addiction (Chen et al., 2017). Studies have suggested that males score higher than females in their locus of control and that both groups differ in their learning styles, which may impact their levels of learning dedication and objective achievement (Khan and Iqbal, 2014; Wehrwein et al., 2015). Additionally, medical students with leadership experience have better self-awareness, self-planning, self-execution, self-assessment, and self-correction skills (Dan, 2013) and serve as role models to other students. Therefore, they need to have better academic achievement and thereby have a low level of academic procrastination (Yao et al., 2021). Understanding the influence of demographics on mobile phone addiction, academic procrastination, and academic achievement can inform interventions and policies aimed at medical students to reduce their mobile phone addiction and academic procrastination.

### Impact of Mobile Phone Addiction on Academic Achievement

In this study, academic achievement was divided into four dimensions. Learning performance examined students’ completion of learning; relationship facilitation assessed students’ interpersonal communication ability; learning dedication focused on students’ enthusiasm and initiative for learning; and objective achievement involved the self-evaluation of moral education, intellectual education, arts and sports, and comprehensive achievements (Yanfei et al., 2011). The four dimensions of academic achievement are considered to accurately assess medical students’ learning consequences, learning attitude, and learning behaviors under medical education standards in China (Li et al., 2020). Our results revealed that mobile phone addiction has a negative impact on learning dedication, learning performance, and relationship facilitation. One study posited that a relationship exists between mobile phone use and academic achievement among university students (Ahmed et al., 2020). Another study involving college students in Hainan showed a 40.5% mobile phone addiction rate (Yan et al., 2017). Similarly, in our survey of medical students in Northeast China, we found that the problem of mobile phone addiction was widespread (Yan et al., 2017). Therefore, Chinese medical students with mobile phone addiction were likelier to report problems in learning dedication, learning performance, and relationship facilitation. Further, incorrect and excessive mobile phone use may lead to a higher risk of depression (Alhassan et al., 2018), poor sleep quality (Liu et al., 2017), loneliness (Li et al., 2016), and academic burnout (Ma et al., 2020). This negatively impacts medical students’ learning and life, and affects their learning dedication, learning performance, and relationship facilitation. However, our study also found that mobile phone addiction did not affect objective achievement. This may be because mobile phones are regarded as study tools that are used to access course materials, search for library catalogs, discuss course assignments with peers, take notes, and so on. Moreover, as the use of mobile phones is often closely related to college studies, students may believe that there is little or no correlation between mobile phone use and objective achievement (Dukic et al., 2015).

### Impact of Academic Procrastination on Academic Achievement

Our results also confirm that academic procrastination has a significant negative influence on academic achievement. This is consistent with the findings of previous studies (Steel and Ones, 2002; Karatas, 2015). Procrastination is a negative defense mechanism that is characterized by escaping or postponing learning tasks (Hoare, 1986). During the process of studying, procrastination may lead to academic failure, and chronic procrastination can cause negative emotions such as tiredness, anxiety, guilt, among others (Ferrari, 2010). Therefore, once medical students show signs of procrastination, it may directly, passively impact their learning dedication and learning performance. However, procrastinators suffer from persistent anxiety about completing tasks, which can lead to other negative emotional reactions; thus, relationship facilitation is affected to some extent (Ferrari et al., 2009). Consequently, educators and teachers should focus on the negative effects of medical students’ academic procrastination.

### Implications for Medical Education

Instructors should consider their students’ demographic factors in addressing mobile phone addiction and academic procrastination among medical students, and accordingly suggest interventions such as cognitive appraisals. Cognitive appraisals can provide insight into medical students’ mobile phone addiction and academic procrastination and can be used by students and educators alike (Ann et al., 2019). Instructors can also guide medical students to learn and practice reducing their academic procrastination, and have regular face-to-face conversations with students who are addicted to their mobile phones (Yanting et al., 2018).

### Limitations

There are several limitations in this study. First, the participants were from four medical schools in the same Chinese province, which may limit the generalizability of this study to other regions. Additionally, we used several scales that were developed for use among Western cohorts, requiring additional academic attention in the Chinese context. Finally, this cross-sectional study reveals the relationship between mobile phone addiction, academic procrastination, and academic achievement at one point, but does not explain the causal relationship between the variables.

## Conclusion

This study revealed that the problems of mobile phone addiction and academic procrastination are prevalent among Chinese medical students, and these negatively influences their academic achievement. Based on these results, we offer guidance for reducing the negative effects of mobile phone addiction and academic procrastination on academic achievement. Future studies are required to identify the factors associated with mobile phone addiction and academic procrastination.

## Data Availability Statement

The original contributions presented in the study are included in the article/supplementary material, further inquiries can be directed to the corresponding author/s.

## Author Contributions

D-PC, S-EZ, and JT contributed to conception and design of the study. J-MX and S-EZ organized the database. Q-LL, C-XZ, and H-CG performed the statistical analysis. J-MX, J-YZ, and JT wrote the first draft of the manuscript. L-BY, L-YZ, and RG wrote sections of the manuscript. All authors contributed to manuscript revision, read, and approved the submitted version.

## Conflict of Interest

The authors declare that the research was conducted in the absence of any commercial or financial relationships that could be construed as a potential conflict of interest.

## Publisher’s Note

All claims expressed in this article are solely those of the authors and do not necessarily represent those of their affiliated organizations, or those of the publisher, the editors and the reviewers. Any product that may be evaluated in this article, or claim that may be made by its manufacturer, is not guaranteed or endorsed by the publisher.

## Footnotes

1
http://www.cnnic.net.cn/hlwfzyj/hlwxzbg/hlwtjbg/

2
http://www.wenjuan.com/
